# Supplementary material for: Sex Differences in Survival from Neuroendocrine Neoplasia in England 2012–2018: A Retrospective, Population-Based Study
Source: Cancers (Basel). 2023 Mar 20;15(6):1863. doi: 10.3390/cancers15061863 (PMC10046836; doi:10.3390/cancers15061863)
Supplement: Supplementary file 1 [file cancers-15-01863-s001.zip › cancers-2046498-supplementary.pdf]

Supplementary Table S1. Sex differential of NEN by main organ primary, stage and morphology. n, number of tumours; NET, neuroendocrine tumour; NEC, neuroendocrine carcinoma

		**Site, n**	**Appendix, n=2,146**							
		**Stage**	**I**		**II**		**III**		**IV**	
		**Morph**	**NET**	**NEC**	**NET**	**NEC**	**NET**	**NEC**	**NET**	**NEC**
**Sex**	**Male**	**n**	453	19	247	12	71	4	16	9
		%	41.0%	57.6%	35.1%	75.0%	32.6%	36.4%	38.1%	50.0%
	**Female**	**n**	652	14	456	4	147	7	26	9
		%	59.0%	42.4%	64.9%	25.0%	67.4%	63.6%	61.9%	50.0%

		**Site, n**	**Caecum, n=528**							
		**Stage**	**I**		**II**		**III**		**IV**	
		**Morph**	**NET**	**NEC**	**NET**	**NEC**	**NET**	**NEC**	**NET**	**NEC**
**Sex**	**Male**	**n**	3	1	8	4	75	23	72	42
		%	30.0%	50.0%	38.1%	44.4%	44.1%	48.9%	41.6%	43.8%
	**Female**	**n**	7	1	13	5	95	24	101	54
		%	70.0%	50.0%	61.9%	55.6%	55.9%	51.1%	58.4%	56.3%

		**Site, n**	**Colon, n=509**							
		**Stage**	**I**		**II**		**III**		**IV**	
		**Morph**	**NET**	**NEC**	**NET**	**NEC**	**NET**	**NEC**	**NET**	**NEC**
**Sex**	**Male**	**n**	25	4	5	10	28	43	59	124
		%	61.0%	80.0%	41.7%	47.6%	60.9%	64.2%	62.8%	55.6%
	**Female**	**n**	16	1	7	11	18	24	35	99
		%	39.0%	20.0%	58.3%	52.4%	39.1%	35.8%	37.2%	44.4%

		**Site, n**	**Lung, n=4,661**							
		**Stage**	**I**		**II**		**III**		**IV**	
		**Morph**	**NET**	**NEC**	**NET**	**NEC**	**NET**	**NEC**	**NET**	**NEC**
**Sex**	**Male**	**n**	660	119	132	46	84	105	167	541
		%	31.3%	38.5%	40.1%	47.4%	39.3%	52.0%	44.9%	52.5%
	**Female**	**n**	1448	190	197	51	130	97	205	489
		%	68.7%	61.5%	59.9%	52.6%	60.7%	48.0%	55.1%	47.5%

		**Site, n**	**Pancreas, n=2,183**							
		**Stage**	**I**		**II**		**III**		**IV**	
		**Morph**	**NET**	**NEC**	**NET**	**NEC**	**NET**	**NEC**	**NET**	**NEC**
**Sex**	**Male**	**n**	229	18	186	26	100	34	320	317
		%	52.2%	32.1%	53.0%	72.2%	57.8%	73.9%	55.2%	63.1%
	**Female**	**n**	210	38	165	10	73	12	260	185
		%	47.8%	67.9%	47.0%	27.8%	42.2%	26.1%	44.8%	36.9%

**Site, n**	**Rectum, n=948**							
**Stage**	**I**		**II**		**III**		**IV**	

		Morph	NET	NEC	NET	NEC	NET	NEC	NET	NEC
**Sex**	**Male**	**n**	275	12	15	12	14	41	27	148
		%	52.9%	57.1%	55.6%	63.2%	56.0%	64.1%	62.8%	64.6%
	**Female**	**n**	245	9	12	7	11	23	16	81
		%	47.1%	42.9%	44.4%	36.8%	44.0%	35.9%	37.2%	35.4%

		Site, n	Small intestine, n=3,201							
		**Stage**	**I**		**II**		**III**		**IV**	
		**Morph**	**NET**	**NEC**	**NET**	**NEC**	**NET**	**NEC**	**NET**	**NEC**
**Sex**	**Male**	**n**	103	1	167	8	710	29	676	110
		%	50.2%	20.0%	63.5%	72.7%	57.0%	58.0%	55.6%	53.7%
	**Female**	**n**	102	4	96	3	536	21	540	95
		%	49.8%	80.0%	36.5%	27.3%	43.0%	42.0%	44.4%	46.3%

		Site, n	Stomach, n=658							
		**Stage**	**I**		**II**		**III**		**IV**	
		**Morph**	**NET**	**NEC**	**NET**	**NEC**	**NET**	**NEC**	**NET**	**NEC**
**Sex**	**Male**	**n**	70	4	36	15	16	48	44	174
		%	40.5%	50.0%	52.2%	75.0%	64.0%	73.8%	65.7%	75.3%
	**Female**	**n**	103	4	33	5	9	17	23	57
		%	59.5%	50.0%	47.8%	25.0%	36.0%	26.2%	34.3%	24.7%
